# A Long-Term Conserved Satellite DNA That Remains Unexpanded in Several Genomes of Characiformes Fish Is Actively Transcribed

**DOI:** 10.1093/gbe/evab002

**Published:** 2021-01-27

**Authors:** Rodrigo Zeni dos Santos, Rodrigo Milan Calegari, Duílio Mazzoni Zerbinato de Andrade Silva, Francisco J Ruiz-Ruano, Silvana Melo, Claudio Oliveira, Fausto Foresti, Marcela Uliano-Silva, Fábio Porto-Foresti, Ricardo Utsunomia

**Affiliations:** 1 Departamento de Ciências Biológicas, Faculdade de Ciências, Universidade Estadual Paulista, UNESP, Campus de Bauru, Bauru, Sao Paulo, Brazil; 2 Departamento de Biologia Estrutural e Funcional, Instituto de Biociências de Botucatu, Universidade Estadual Paulista, UNESP, Botucatu, Sao Paulo, Brazil; 3 Department of Organismal Biology—Systematic Biology, Evolutionary Biology Centre, Uppsala University, Uppsala, Sweden; 4 Wellcome Sanger Institute, Cambridge, United Kingdom; 5 Departamento de Genética, Instituto de Ciências Biológicas e da Saúde, ICBS, Universidade Federal Rural do Rio de Janeiro, Seropédica, Rio de Janerio, Brazil

**Keywords:** repetitive DNA, neotropical fish, tandem repeats, satDNA

## Abstract

Eukaryotic genomes contain large amounts of repetitive DNA sequences, such as tandemly repeated satellite DNAs (satDNAs). These sequences are highly dynamic and tend to be genus- or species-specific due to their particular evolutionary pathways, although there are few unusual cases of conserved satDNAs over long periods of time. Here, we used multiple approaches to reveal that an satDNA named CharSat01-52 originated in the last common ancestor of Characoidei fish, a superfamily within the Characiformes order, ∼140–78 Ma, whereas its nucleotide composition has remained considerably conserved in several taxa. We show that 14 distantly related species within Characoidei share the presence of this satDNA, which is highly amplified and clustered in subtelomeric regions in a single species (*Characidium gomesi*), while remained organized as small clusters in all the other species. Defying predictions of the molecular drive of satellite evolution, CharSat01-52 shows similar values of intra- and interspecific divergence. Although we did not provide evidence for a specific functional role of CharSat01-52, its transcriptional activity was demonstrated in different species. In addition, we identified short tandem arrays of CharSat01-52 embedded within single-molecule real-time long reads of *Astyanax paranae* (536 bp–3.1 kb) and *A. mexicanus* (501 bp–3.9 kb). Such arrays consisted of head-to-tail repeats and could be found interspersed with other sequences, inverted sequences, or neighbored by other satellites. Our results provide a detailed characterization of an old and conserved satDNA, challenging general predictions of satDNA evolution.

**Significance**

The genomes of eukaryotes are significantly composed by noncoding repeated DNA sequences, known as satellite DNAs (satDNAs). In general, these sequences have no defined function and represent a fast-evolving portion of the genome. For this reason, these sequences are usually species or genus specific and the evolutionary persistence of these sequences over a long period is uncommon and not well understood. Here, we found a highly conserved satellite that originated 140–78 Ma and persisted in the genomes of several fish species in an entire order. By using multiple approaches, we showed that this sequence remained as a typical satDNA in all species and is actively transcribed. Here, we provide possible explanations for the long-term maintenance of this satDNA.

## Introduction

Satellite DNAs (satDNAs) are noncoding tandemly repeated sequences that constitute large portions of eukaryotic genomes, with head-to-tail arrays reaching up to hundreds of thousands of nucleotides (López-Flores and Garrido-Ramos 2012; Plohl et al. 2012). These sequences are preferably found on the heterochromatin of pericentromeric and subtelomeric regions, although their occurrence in euchromatic areas has been reported (Plohl et al. 2012; Garrido-Ramos 2015; Ruiz-Ruano et al. 2016; Silva et al. 2017). In general, it is assumed that satDNAs originate de novo from random duplication events of a genomic sequence of two or more nucleotides that spread throughout the genome by distinct mechanisms, such as multiple transposable element insertions and/or rolling circle replication and reinsertion (Ruiz-Ruano et al. 2016; Vondrak et al. 2020). Afterward, stochastic events may lead to the local amplification of those short arrays or to their extinction in the referred locus/genome (Plohl et al. 2012; Ruiz-Ruano et al. 2016; Lower et al. 2018). Remarkably, every satDNA locus within a genome will transcend speciation events and evolve independently in each lineage, giving rise to the library hypothesis model of satellite evolution, which predicts that related species share a common collection of satDNAs that may be independently amplified or depleted over time (Fry and Salser 1977).

Although highly repetitive and usually spread throughout different chromosomes and/or genomic regions, satDNAs usually exhibit high intraspecific repeat homogeneity and interspecific heterogeneity, which is related to the concerted evolution of these satellite repeats, reached by intraspecific sequence homogenization and fixation (Dover 1982,  1986). In the context of concerted evolution and the general absence of functional selective constraints, satDNA sequences are frequently reported as being species or genus specific, with few examples of satellite repeats being conserved over a long period of time (e.g., more than 50 Myr) (Plohl et al. 2012; Lorite et al. 2017; Halbach et al. 2020).

The order Characiformes is a species-rich clade in the tree of life, with representatives restricted to freshwater environments of Africa and the Americas (Betancur-R et al. 2019). This group is split into two well-characterized monophyletic suborders: Citharinoidei, with ∼110 species in two families, and Characoidei, with almost 2,000 species in 22 families (Arcila et al. 2017; Chakrabarty et al. 2017; Dai et al. 2018; Hughes et al. 2018; Betancur-R et al. 2019). The accumulated cytogenetic data for this group include great karyotype diversification, distinct sex chromosome systems, independent origins of supernumerary chromosomes, and multiple cases of repetitive DNA sequence diversification, notably, multigene families (Oliveira et al. 2009; Cioffi et al. 2011). On the other hand, satDNA information is mainly restricted to unique or few species from the same family (Vicari et al. 2010).

In recent years, powered by the expansion of next-generation sequencing and bioinformatic protocols, entire collections of satDNAs have been described for several species, mainly invertebrates and fishes (Ruiz-Ruano et al. 2016; Palacios-Gimenez et al. 2017; Pita et al. 2017; Silva et al. 2017; Utsunomia et al. 2019; Serrano-Freitas et al. 2020). Remarkably, satellitome analyses performed by us within the Characiformes fish, including distinct species belonging to the Crenuchidae, Anostomidae, and Characidae families, revealed the existence of a conserved 52-bp-long satDNA (CgomSat02-52, ApaSat29-52, and MmaSat85-52), named here CharSat01-52. Considering that Crenuchidae is a sister group of most Characiformes (Arcila et al. 2017; Betancur-R et al. 2019), an initial hypothesis of the long-term existence of an satDNA family has been proposed (Utsunomia et al. 2019).

Here, we delimited the origin and assessed the genomic organization of this ancient satDNA among Characiformes by analyzing short-read data from 14 species encompassing nine families within this order—Distichodontidae, Crenuchidae, Erythrinidae, Hemiodontidae, Serrasalmidae, Prochilodontidae, Anostomidae, Bryconidae, and Characidae—that diverged more than 100 Ma (Arcila et al. 2017; Hughes et al. 2018). Furthermore, fluorescent in situ hybridization (FISH) experiments were performed and corroborated the in silico analyses, evidencing that the clustered pattern is restricted to a single species. Next, we used long-read data (PacBio sequencing) to decipher the lengths and densities of CharSat01-52 in two species exhibiting a nonclustered pattern and compared them against those of a clustered satDNA. The resulting data suggest the long-term maintenance of CharSat01-52, which mainly experienced quantitative changes among species, for dozens of millions of years, corroborating the library hypothesis. However, extreme sequence conservation also defies predictions of concerted evolution patterns.

## Results

### Repeat Identification and Intra- and Interspecific Abundance and Divergence Values

**Fig. 1:**
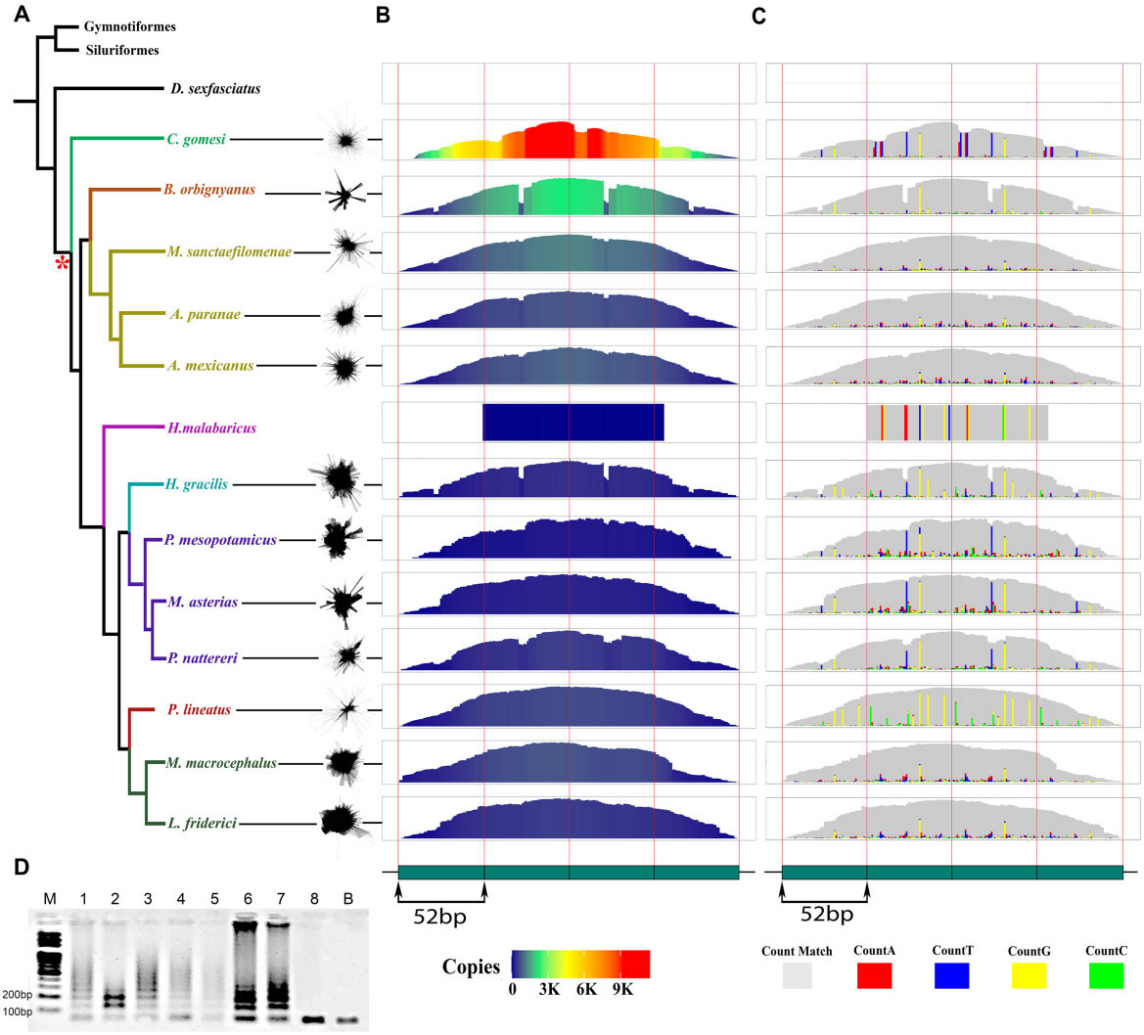
A head-to-tail tandem repeat organization of CharSat01-52 occur widely in several Characiformes species. (*A*) Phylogenetic tree adapted from Betancur-R et al. (2019) showing the relationships between the Characiformes species analyzed here. Colors in the clade indicate distinct families within this order (Distichodontidae, Crenuchidae, Bryconidae, Characidae, Erythrinidae, Hemiodontidae, Serrasalmidae, Prochilodontidae, and Anostomidae). On the right side of each species, the outputted graphs after clustering reads with circular shapes. Red asterisk denotes the proposed origin of CharSat01-52, restricted to Characoidea species. (*B*) CNV profiles for CharSat01-52. Note the higher abundance of the referred satDNA in *Characidium gomesi*. (*C*) Variant profiles for CharSat01-52 against a consensus sequence. Note the similarity of profiles in the within-family level. **(***D*) Agarose gel electrophoresis after PCR amplification in several species. M: molecular marker, 1: *Astyanax paranae*, 2: *Moenkhausia sanctaefilomenae*, 3: *Brycon orbignyanus*, 4: *Leporinus friderici*, 5: *Megaleporinus macrocephalus*, 6: *Prochilodus lineatus*, 7: *C. gomesi*, 8: *Hoplias malabaricus*, B: negative control.

To delimit the occurrence of CharSat01-52, we searched for this satDNA in the genomes of several fish species by using multiple approaches. BLAT searches followed by graph clustering with RepeatExplorer generated sphere-shaped graphs for all the Characoidei genomes analyzed, except that of *Hopilas malabaricus*. This indicates that CharSat01-52 is consistently present as a typical satDNA in the referred species (fig. 1*A*). We retrieved 52-bp-long monomers from all the species and calculated the A + T content of the consensus sequences. This was biased toward A + T richness, varying from 59.3% to 71.5%, with a median value of 65.2%.

The CharSat01-52 copy number verification (CNV) profiles indicated that this satellite shows a higher abundance in *Characidium gomesi* (average coverage = 2,697 copies, with a peak at 12,000 copies) than in the other species, in which CharSat01-52 abundance seems to be lower (mean = 181 copies, standard deviation [SD] = 712.8; table 1, fig. 1*B*, and supplementary fig. S1, Supplementary Material online). All the obtained scaled profiles showed high correlation values among each other (*r* = 0.99), pointing to a conserved, tandemly arrayed monomer structure in all the species, except *H. malabaricus*. Notably, a 3-bp valley (monomer positions 22–24) was observed in the graphs of *Brycon orbignyanus* and *Hemiodus gracilis*, indicating a deletion of these bases in approximately half of the copies of CharSat01-52 in both genomes (fig. 1*B*). In general, the variant profile graphs were similar among species belonging to the same families (fig. 1*C*), consistent with the phylogenetic relationships. Additionally, some recurrent variants were observed, such as position 32 of the CharSat01-52 monomers, which seem to be prone to variation in all the analyzed species (fig. 1*C*).

**Table 1:** Main Results of CNV Profile of CharSat01-52 in Multiple Characiformes Species

Species	Total Reads	Proportion of Bases with Coverage	Normalized Average Coverage
*Distichodus sexfasciatus*	5,000,000	0	0
*Characidium gomesi*	5,000,000	0.975961538	2,822.325499
*Characidium gomesi*	5,000,000	1	2,876.667544
*Characidium gomesi*	5,000,000	0.975961538	2,697.887615
*Brycon orbignyanus*	5,000,000	1	554.1420943
*Moenkhausia* *sanctaefilomenae*	5,000,000	1	326.909913
*Astyanax paranae*	5,000,000	1	205.7046328
*Astyanax mexicanus*	5,000,000	1	245.5193184
*Hopilas malabaricus*	5,000,000	0.533653846	1.536351898
*Hemiodus gracilis*	4,173,860	1	95.39636771
*Piaractus mesopotamicus*	4,914,670	0.9375	43.55158902
*Myleus asterias*	5,000,000	1	68.52400619
*Pygocentrus nattereri*	5,000,000	0.995192308	129.9237403
*Prochilodus lineatus*	5,000,000	1	178.0744875
*Megaleporinus* *macrocephalus*	2,318,964	1	202.8145728
*Leporinus friderici*	2,235,104	0.990384615	131.5511865

The repeat landscapes also pointed to a higher abundance of CharSat01-52 in *C. gomesi* and evidenced some distinctive species-specific or family specific landscape shapes (table 2 and supplementary fig. S2, Supplementary Material online), corroborating the variant profiles results. For example, the landscapes obtained from *C. gomesi*, *M*egaleporinus *macrocephalus*, *Prochilodus lineatus*, *B. orbignyanus*, and *Moenkhausiasanctaefilomenae* each exhibited a different peak of abundance in particular Kimura divergence values, pointing to a differential amplification of variants in each species (supplementary fig. S2, Supplementary Material online). Notably, very similar landscape patterns, with small intraspecific deviations, were retrieved by analyzing multiple individuals of *C. gomesi* (supplementary fig. S2, Supplementary Material online and table 2), indicating a low degree of interindividual variation. Remarkably, none of the approaches applied here were capable of detecting signals of CharSat01-52 presence in the genomes of the non-Characoidei species *Distichodus sexfasciatus*, *G. sylvius*, and *P. corruscans*. On the other hand, although we were not able to collect CharSat01-52 monomers from the *H. malabaricus* genome, two reads were isolated using RepeatMasker and RepeatProfiler (fig. 1*B*), suggesting the residual existence of this satDNA in this species.

**Table 2:** Genetic Variation and Main Features of CharSat01-52 Obtained from DNA-seq Data

Species	*N*	Monomer Size (bp)	A + T (%)	Abundance (%)	Intraspecific KD (%)	Interespecific KD (%)
*Characidium gomesi*	2,145	52	71.5	3.53E−03 ± 9.69E−05	15.43 ± 0.03	
*Hopilas malabaricus*	—	52		3.03917E−05	2.15E+01	
*Hemiodus gracilis*	116	52	66	0.000923	1.61E+01	
*Piaractus mesopotamicus*	6	52	67.4	0.000124	1.94E+01	
*Myleus asterias*	12	52	66.2	0.000155	1.82E+01	
*Pygocentrus nattereri*	63	52	65.5	0.000182849	1.86E+01	
*Prochilodus lineatus*	90	52	59.3	0.000287	1.44E+01	
*Leporinus friderici*	69	52	67.4	0.000196	1.45E+01	
*Megaleporinus* *macrocephalus*	187	52	67.6	0.000315	1.35E+01	
*Brycon orbignyanus*	63	52	64.6	0.000191	8.16E+00	
*Astyanax mexicanus*	33	52	68.2	0.000179	1.32E+01	
*Astyanax paranae*	65	52	67.7	0.000137	1.13E+01	
*Moenkhausia* *sanctaefilomenae*	104	52	67.9	0.00074	9.48E+00	
*Distichodus sexfasciatus*	—	—	—	0	0.00E+00	
Total	2,953					15.22
Mean			65.2	0.000499303	1,37E+01	
SD			4.020779361	0.000909406	5.47E+00	
Coefficiente of variation			6.16683951	182.135036	39.51418598	

**Fig. 2:**
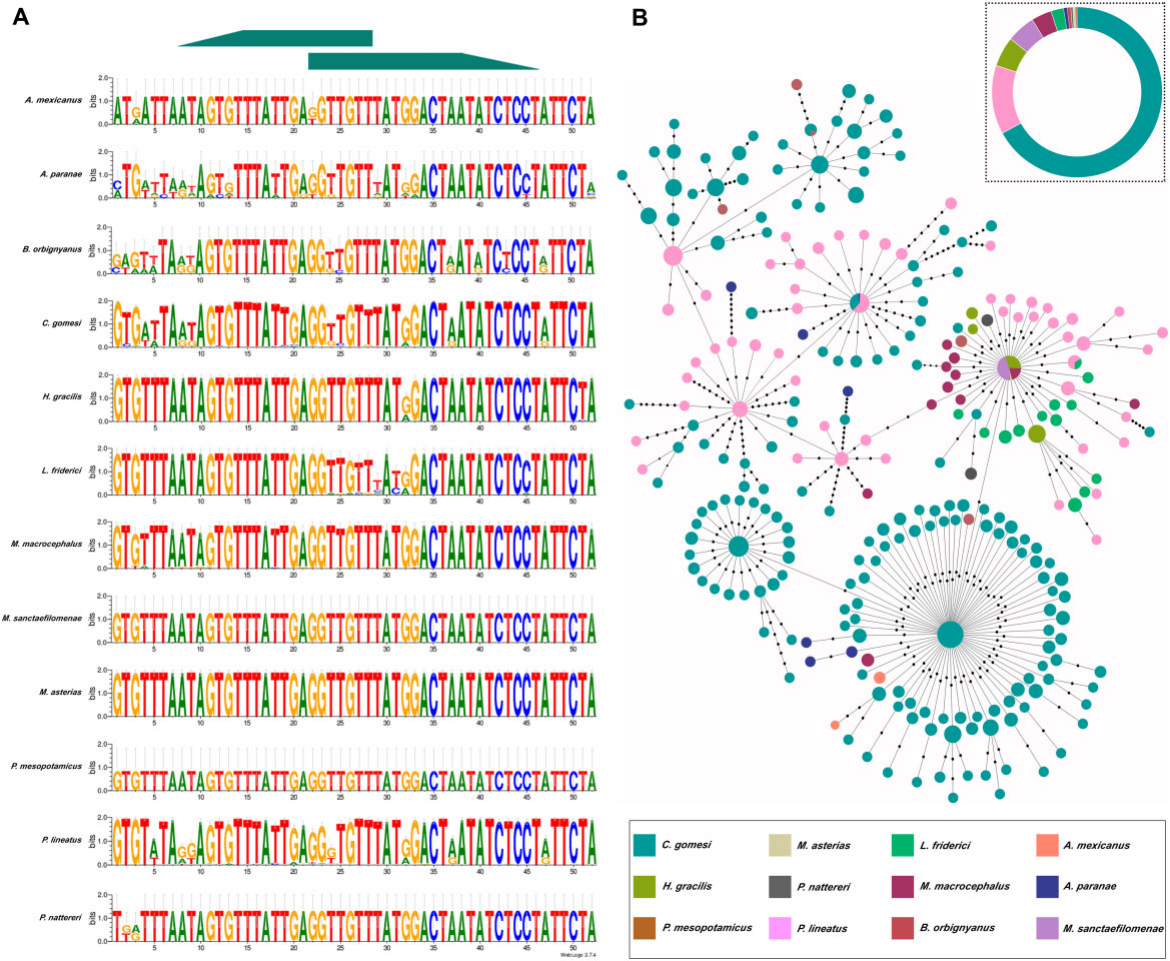
**—(** *A*) CharSat01-52 sequence logos denoting a considerable sequence conservation of this satellite DNA. Green arrows indicate the anchoring regions of primers previously designed by Silva et al. (2017). (*B*) MST showing the relationships between the isolated monomers obtained from distinct species. Colored circles represent monomers retrieved from Illumina reads and the diameter of the circles is proportional (log scale) to their abundance. Each black dot represents a mutational step. Note the multiple occurrences of shared variants, including one common to five species. **(***C*) Quantification of relative copy number of CharSat01-52 in several species.

Direct short read–derived monomer extraction was performed for all the species and the resulting data were aligned to generate separate sequence logos, which indicated a general intra- and interspecific conservation of this satDNA, with particular positions exhibiting polymorphisms (table 2, fig. 2, and supplementary fig. S3, Supplementary Material online), as evidenced in the analysis of the variant profile coverage. After a global alignment, we produced a minimum spanning tree (MST) that depicted an interesting scenario for CharSat01-52, since general species-specific groups of haplotypes were not a general rule, except for some grouped haplotypes of *C. gomesi* (fig. 2 and supplementary fig. S3, Supplementary Material online). Additionally, several variants (haplotypes) were shared among distantly related species, including two variants that were common to three and five species. Notably, these shared variants do not reflect the phylogenetic proximity between the referred species (fig. 2 and supplementary fig. S3, Supplementary Material online). The interspecific Kimura divergence (KD) value obtained here was similar or even higher than the intraspecific values (table 2), corroborating the MST results. Quantification of relative copy number of CharSat01-52 was investigated by quantitative polymerase chain reaction (qPCR) and results obtained confirmed a higher abundance of this sequence in *C. gomesi*, as expected (fig. 2).

BLAST searches of the CharSat01-52 consensus sequence against the nucleotide collection of the NCBI produced different significant alignments. As expected, low *e*-values (max *e*-value = 1e−10) were observed for the previously described variants (MmaSat085-52, MelSat49-52, ApaSat29-52, and CgomSat02-52). In addition, significant matches (*e*-value* *=* *2e−08) with transcript variants of the PTPRF interacting protein alpha 1 (*ppfia1*) from *Astyanax mexicanus* were also obtained. Downstream analyses of the assembled genomes of several Ostariophysi species (*A. mexicanus*, *Pygocentrus nattereri*, *Pangasianodon hypophthalmus*, *Electrophorus electricus*, and *Danio rerio*) revealed the occurrence of a CharSat01-52 array (34 imperfect monomers reaching ∼1,809 bp) near the end of the 3′-UTR region of this gene in *A. mexicanus.* After that, we manually searched the same region in the *P. nattereri* genome and obtained similar results, with the occurrence of an array of 23 imperfect monomers of CharSat01-52 reaching ∼1,263 bp located 637 bp downstream of the corresponding exon (supplementary fig. S4, Supplementary Material online). Although the position of the CharSat01-52 array is similar in both species, the *ppfia1* gene has an additional exon in *P. nattereri* in comparison with that in *A. mexicanus* (supplementary fig. S4, Supplementary Material online). For this reason, the satDNA array is located within the last intron in *P. nattereri*.These results elucidate the positive BLAST search for CharSat01-52 for only the *ppfia1* of *A. mexicanus*, since one perfect monomer appears to be transcribed in this species as a part of the 3′-UTR, which is not the case for *P. nattereri*. Considering the other analyzed species belonging to distinct orders, we could not find any tandemly repeated pattern of sequences in the corresponding regions of this gene (supplementary fig. S4, Supplementary Material online).

### CharSat01-52 Is Tandemly Repeated in Several Genomes—PCR and FISH

**Fig. 3:**
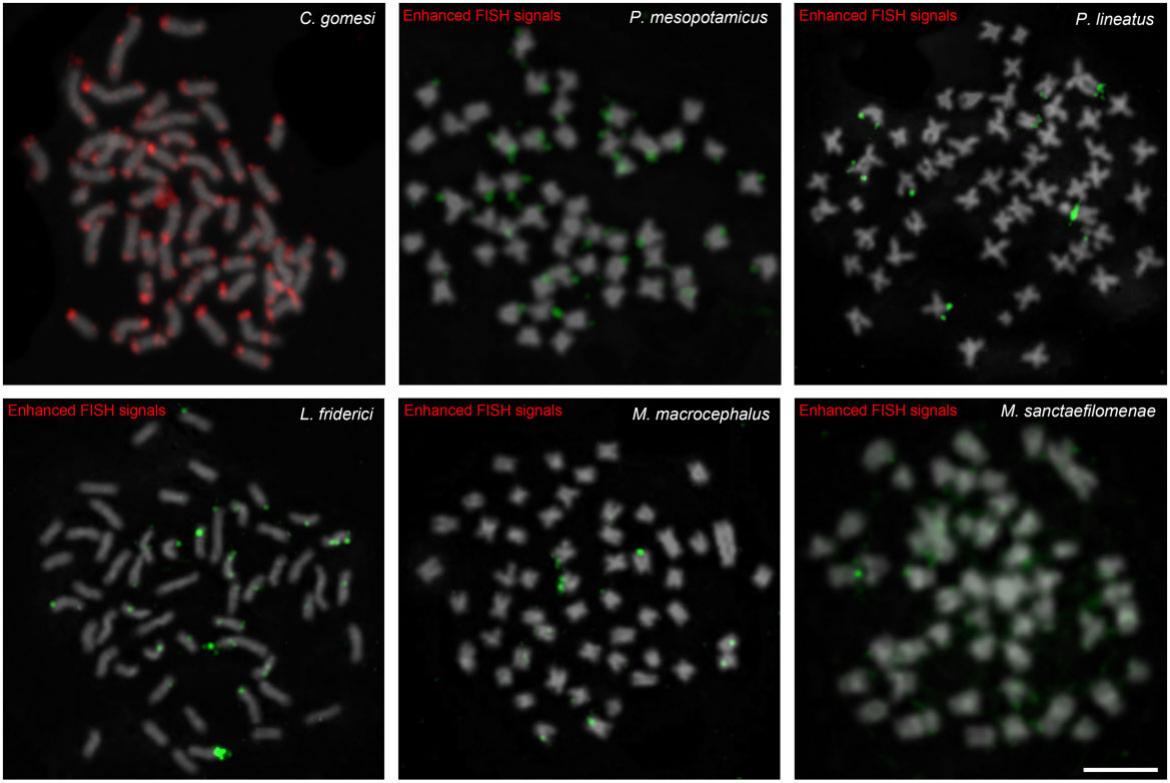
Distribution of CharSat01-52 on the metaphase chromosomes of several Characoidea species. Note that two rounds of signal enhancement were carried out in all species, except *Characidium gomesi*, indicating that large clusters are only present in *C. gomesi*. Bar =10 μm.

Polymerase chain reaction (PCR) amplification of the referred satDNA in eight species corroborated the in silico analyses and yielded a ladder-like pattern of bands in all the species, except *H. malabaricus* (fig. 1*D*). After that, the FISH probes labeled with digoxigenin-dUTP were hybridized against the chromosomes of eight species within the Characiformes, which yielded visible FISH signals only in *C. gomesi*, in which it displays intense signals on subtelomeric regions of all chromosomes (fig. 3). All the other analyzed species did not show primarily any visible signals (supplementary fig. S5, Supplementary Material online), probably as a result of the CharSat01-52 sequences being organized in short arrays, that is, <10 kb, the boundary of the sensitivity of the FISH technique, in these species, as we further confirmed with long-read data (see below). After enhancing the FISH signals of CharSat01-52 using conjugated antiavidin-biotin, we confirmed that this satDNA is organized as short tandem arrays in all species (figs. 3 and 5).

### Transcription of CharSat01-52

**Fig. 4:**
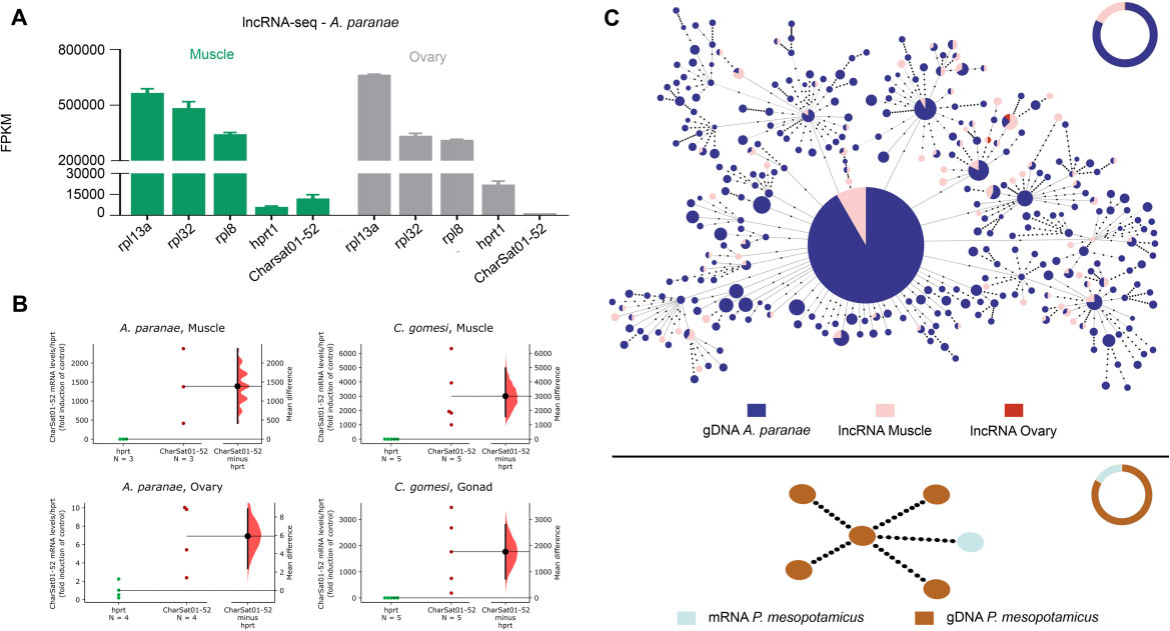
**—**Transcription analysis of CharSat01-52. (*A*) Transcription levels of CharSat01-52 and several other endogenous genes in different lncRNA-seq libraries from *Astyanax paranae*, measured as FPKM. (*B*) Gardner-Altman estimation plots showing CharSat01-52 transcription levels in gonads and muscle tissues of *A*. *paranae* and *Characidium gomesi* individuals, analyzed by RT-qPCR. Both groups are plotted on the left axes and the mean difference (effect size) is plotted on a floating axe on the right as a bootstrap sampling distribution. The mean difference is depicted as a black dot, and the 95% confidence interval is indicated by the ends of the vertical error bar. (*C*) MSTs showing the relationships between the isolated monomers from gDNA-seq and RNA-seq libraries. Note that the most abundant variant in the genome of *A. paranae* is also the most transcribed variant.

The expression analysis revealed that CharSat01-52 is expressed in the muscle and ovaries of *A. paranae* as well as in the muscle of *Piaractus mesopotamicus* (fig. 4). Importantly, the read counts of CharSat01-52 were directly affected by and associated with the protocol applied to generate the RNA sequencing (RNA-seq) libraries (i.e., the lncRNA or mRNA libraries). The expression of CharSat01-52 in the lncRNA libraries was ∼15.4 times higher in the muscle than in the ovaries of *A. paranae* (*P* = 0.0022, *t* = 5.71, df = 5.035; fig. 4). In the mRNA libraries, the expression was 4.5 times higher in the muscle than in the ovaries (*P* = 0.0001, *t* = 6.171, df = 10; supplementary fig. S6, Supplementary Material online).

The generated MST from monomers derived from DNA- and RNA-seq libraries revealed an interesting divergence of transcribed monomers in different tissues of *A. paranae* and *P. mesopotamicus*. Notably, the most abundant RNA-seq-derived monomer of *A. paranae*, which is a monomer shared with *C. gomesi* and *P. lineatus*, is the most abundant variant in the gDNA-derived sequences (figs. 2 and 4). We also performed RT-qPCR in different tissues of *A. paranae* and *C. gomesi*. Results obtained for *A. paranae* corroborated the RNA-seq data, with a higher expression of CharSat01-52 in the muscle compared with the ovaries. For *C. gomesi*, we observed that this satDNA is also transcribed in both tissues, with a higher expression in the muscle (fig. 4).

### Estimating CharSat01-52 Repeat Abundance and Array Sizes Using PacBio SMRT Reads

**Fig. 5:**
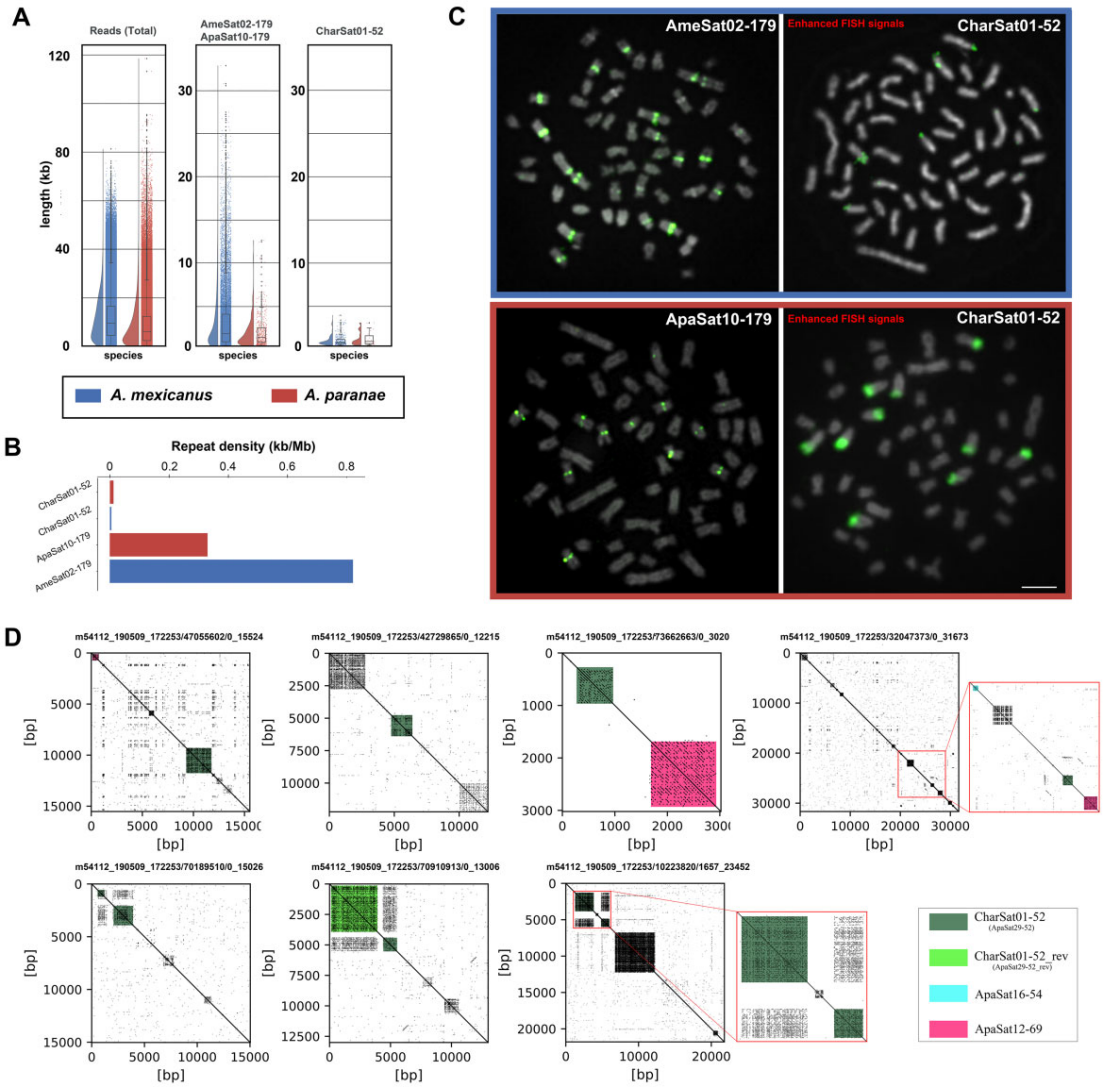
(*A*) Raincloud plots of lengths of reads, AmeSat02-179/ApaSat10-179 and CharSat01-52, recovered from PacBio data of *Astyanax paranae* and *A. mexicanus*. (*B*) Overall repeat density of CharSat01 and AmeSat02-179/ApaSat10-179. (*C***)** Metaphase plates after FISH with distinct probes, as indicated in the figure. Metaphases bordered in blue are from *A. mexicanus*, whereas metaphases bordered in red are from *A. paranae*. (*D*) Annotated dot plots showing isolated cases of CharSat01-52 arrays interspersed with other sequences, inverted sequences or neighbored by other satellites, revealing that ChatSat01-52 arrays are not always consisted of perfect head-to-tail monomers. Bar =10 μm.

Overall, the throughput of the PacBio sequencing subreads of *A. paranae* was 3.04 Gb, whereas the downloaded data for *A. mexicanus* totaled 28.5 Gb (fig. 5). The calculated repeat densities for each satDNA in the single-molecule real-time (SMRT) reads showed that the AmeSat02-179/ApaSat10-179 (clustered satDNA) density was 27.2- and 174.4-fold higher than the CharSat01-52 (nonclustered satDNA) density in *A. paranae* and *A. mexicanus*, respectively, corroborating the in silico and FISH analyses. The lengths of the arrays were also consistent with the FISH results (fig. 5). Thus, the longest repeat arrays we recovered for the highly clustered satellites, that is, those detected by the FISH experiments without signal enhancement (AmeSat02-179/ApaSat10-179), were over 32.8 and 12.6 kb in *A. mexicanus* and *A. paranae*, respectively. Conversely, the FISH signals for CharSat01-52 were visible exclusively after signal enhancement (see Materials and Methods), corroborating the in silico analyses that evidenced that the longest arrays of this satDNA were 3.9 and 3.1 kb. Finally, our two approaches to identify the recurrent association of sequences with CharSat01-52 arrays did not return any associated sequence with this satDNA in our libraries. Thus, although specific and isolated cases of association between CharSat01-52 and other described satDNAs were found, we did not find a recurrent association between CharSat01-52 with other sequence.

## Discussion

In this study, we identified and characterized a conserved satDNA in 14 Characoidea species using multiple approaches and dissected the array organization of this satDNA by using long reads from two species. CharSat01-52 exhibits the main features of an authentic tandem repeat in almost all the sampled Characoidea species, as evidenced by the ladder-like pattern of PCR amplification and the tandem-repeated structure of RepeatExplorer contigs (Novák et al. 2013). Given the occurrence and distribution of CharSat01-52, we suggest that this satDNA originated in the last common ancestor species of Characoidea, before the split of the Crenuchidae (*C. gomesi*), which lived ∼140–78 Ma (Burns and Sidlauskas 2019; Melo personal communication). To our knowledge, this is one of the oldest satDNA sequences described so far, along with APSP-I in ants (80–74 Ma; Lorite et al. 2017), PRAT in coleopterans (60–50 Ma; Mravinac et al. 2002), PstI in sturgeon fishes (100 Ma; Robles et al. 2004), and the three most ancient satDNA sequences ever reported: BIV160 and PjHaaI in molluscs (540 Ma; Plohl et al. 2010; Petraccioli et al. 2015), and tapiR in *Drosophila* (200 Ma; Halbach et al. 2020).

We also combined different sequencing technologies to enhance our knowledge about satDNA array organization, since the genomic analyses of long reads applied to satDNAs have been restricted to highly clustered satellites to date (Khost et al. 2017; Cechova et al. 2019; Heitkam et al. 2020; Vondrak et al. 2020). Here, by analyzing SMRT reads with NoiseCancellingRepeatFinder (NCRF) software (Harris et al. 2019), an algorithm that tackles the noisy error profiles of PacBio and Nanopore reads, we were able to recover several tandemly arrayed CharSat01-52 sequences in two species that did not primarily produce conspicuous cluster-type signals after FISH, unless the signals are enhanced (*A. paranae* and *A. mexicanus*). These arrays did not sum up to 5 kb long in both species, explaining the requirement of signal enhancement to detect FISH signals (as this method has a sensitivity of ∼10 kb). In fact, the array sizes found for a known highly clustered satDNA were much longer in both species (up to 32.8 kb in *A. mexicanus*).

Recent results related to satDNA organization have revealed that, in general, satDNA arrays are usually composed of a mix of perfect and incomplete repeats interspersed by and/or adjacent to different kinds of sequences, including different transposable element families, which usually participate in the spreading of satellites (Khost et al. 2017; Cechova et al. 2019; Heitkam et al. 2020; Vondrak et al. 2020). Our data indicated that CharSat01-52 constitutes small tandem arrays but can also be interspersed by other sequences, as well as adjacent to different known and unknown repetitive DNA sequences, including tandem repeats (fig. 5). However, we could not identify a recurrent common pattern of association between CharSat01-52 and other elements, indicating that specific transposable elements do not seem to actively participate in the intragenomic diversification of this satDNA.

The occurrence of a CharSat01-52 array in different noncoding regions of *ppfia1* (e.g., the intron and 3′-UTR) in two Characiformes species is notable but should not be taken as evidence of its origin, as in the CapA satDNA, present in Platyrrhini mammals, which is suggested to have originated from the intron of the NOS1AP gene (Valeri et al. 2018). In the referred case, the authors found that CapA satDNA is arranged in a single-copy fashion in several eutherian genomes, whereas it is amplified and tandemly arrayed in only the Platyrrhini clade. Here, we did not find any sign of CharSat01-52 presence in other non-Characiformes species or any similarity between this satDNA and other sequences, such as transposable elements or other noncoding sequences. For this reason, we suggest that CharSat01-52 sequences were inserted into the noncoding regions of *ppfia1* after it originated as an satDNA and that this occurred at least before the split of the Characidae (*A. mexicanus*) and Serrasalmidae (*P. nattereri*).

Current ideas of satDNA evolution include the library hypothesis and concerted evolution of repeats (Fry and Salser 1977; Dover 1982). Together, both models can explain the evolution of the great majority of satDNAs described so far, which include high chromosomal and nucleotide dynamics, the occurrence of species- or genus-specific sequences, high levels of intraspecies sequence homogeneity, and low rates of evolutionary persistence (Dover, 1982; Garrido-Ramos 2015, 2017). For this reason, the long-term conservation of satDNAs is unexpected and not yet well understood. After its origin, CharSat01-52 experienced array amplification (e.g., *C. gomesi*) and depletion (e.g., *H. malabaricus*) events, which is consistent with the library hypothesis. Such quantitative changes may be attributed to events like unequal crossing over as well as loop deletions and reinsertion of resulting extrachromosomal circles (Smith 1976; Walsh 1987; Plohl et al. 2008; Lower et al. 2018). Remarkably, the complete depletion of satDNA arrays is a dead end and neutrally evolving arrays will eventually reach this state and become extinct (Charlesworth et al. 1986; Lower et al. 2018).

Previous studies indicated that the rate of recombination in short arrays would be too low to fully homogenize the repeats (Dover 1982; Ambrose and Crease 2011; Pavlek et al. 2015). Here, our data revealed that homogenization of CharSat01-52 repeats is taking place in all the species, regardless of their genomic organization (highly clustered or not). Our data also defy the expectations of molecular drive, since the interspecific KD values were not higher than the intraspecific values (table 2), and several monomer sequences are shared among distantly related species, including one variant present in at least five of them, which does not reflect their phylogenetic relationships.

The satellite landscape profile of a given genome is a multifactorial feature that depends on several components, such as genomic organization and homogenization patterns, population and reproductive issues, and even functional constraints (Dover 1982; Mravinac et al. 2002; Meštrović et al. 2006; Kuhn et al. 2008; Chaves et al. 2017; Smalec et al. 2019). In this context, the existence of long-term conserved satDNA in sturgeon fishes, for example, was explained by a low mutation and homogenization rate (de la Herrán et al. 2001). Slow rates of evolution are not restricted to this single satDNA, but sturgeon genomes as a whole tend to evolve more slowly than those of other teleosts (Du et al. 2020). Here, it does not seem that a general slow evolution could explain the conservation of CharSat01-52, since this is the only satDNA common to all four species from three distinct families within the Characiformes (from a sample of more than 200 satDNA families) (Silva et al. 2017; Utsunomia et al. 2019; Serrano-Freitas et al. 2020; Crepaldi and Parise-Maltempi 2020). However, considering that satDNA families evolve independently within a genome (Kuhn et al. 2008), slow rates of concerted evolution in CharSat01-52 could explain its conservation across millions of years. In fact, a general similarity among samples from the same family in the shapes of the repeat landscapes is observed. Another explanation would be the particular combinations of nucleotides and structural features of the DNA molecule that are favored by homogenization mechanisms or their functional potential, characterizing a selective constraint (Plohl et al. 2008, 2012).

Although the transcriptional activity of satellites can be viewed as a failure of normal transcription termination (e.g., the “read-through” hypothesis, Varley et al. 1980; Epstein et al. 1986; Deryusheva et al. 2007), recent studies have revealed that satDNA transcripts might be involved in several cellular functions and could act as noncoding RNAs, which could explain their evolutionary persistence (Pezer et al. 2012; Petraccioli et al. 2015; Ferreira et al. 2019; Halbach et al. 2020; Louzada et al. 2020). Here, we detected CharSat01-52 transcripts in different tissues of three distantly related species (140–78 Ma) and, whether this satDNA has been actively conserved in Characoidei species through selective constraints or due to other unknown mechanisms remains to be investigated in the near future. Importantly, one must note that the lncRNA libraries retained many more CharSat01-52 fragments than enriched poly-A fragments, suggesting that satDNA transcription should be analyzed from rRNA-depleted total RNA libraries.

In the present study, by using multiple approaches, we delimited the occurrence and origin of a conserved satDNA that remains unexpanded as short arrays in several genomes of Characiformes fish, whereas it became highly abundant in *C. gomesi*. Although intragenomic homogenization was observed, an unusual case of interspecific homogenization was also found, which might be explained by functional constraints, since CharSat01-52 monomers are actively transcribed in distinct tissues of *A. paranae* and *C. gomesi*. Moreover, by analyzing the long reads of two species, we corroborated the recent view that satDNA loci are not homogeneous head-to-tail arrays, as we found several small arrays interspersed with other sequences; however, we did not find evidence of recurrent association with transposable elements, for example. Thus, despite the high error rates (∼15% for subreads), a growing interest in workflows directed at the analysis of satDNAs on raw long reads in the next few years is expected. By combining different technologies, we call attention to the importance of analyzing the genomic structure of repetitive sequences using multiple layers of information.

## Materials and Methods

### Ethics

The animals were collected in accordance with Brazilian environmental protection legislation (Collection Permission MMA/IBAMA/SISBIO—number 3245), and the procedures for the sampling, maintenance and analysis of the fishes were performed in compliance with the Brazilian College of Animal Experimentation (COBEA) and approved (protocol 504) by the Bioscience Institute/UNESP Ethics Committee on the Use of Animals (CEUA).

### Sampling

Here, we analyzed several Characiformes species for distinct purposes. Cell suspensions of some species containing mitotic metaphase plates were already available in our laboratory from previous studies (Silva et al. 2013, 2014, 2016; Scacchetti et al. 2015; Utsunomia et al. 2016; supplementary table S1, Supplementary Material online).

Genomic DNA was extracted from the muscle, liver, or blood of several species and preserved in 100% ethanol using the Wizard Genomic DNA Purification Kit (Promega) following the manufacturer’s instructions, including a step for RNA removal with RNAse A (Invitrogen). The samples were run on 1% agarose gel to check the DNA integrity. Total RNA extraction was performed using the TRIzol Kit (Invitrogen) following the manufacturer’s instructions. Then, the samples were treated with DNAse I (Thermo Fisher Scientific) and checked on 1% agarose gel and with 2100 Bioanalyzer (Agilent) equipment. Only RNA samples with RIN > 7 were used for the subsequent analysis. Information regarding the sampling and methods applied for each specimen is detailed in supplementary table S1, Supplementary Material online.

### Sequencing Data

To uncover the extension and presence of CharSat01-52, we analyzed short-read sequencing data from species comprising three different fish orders within Otophysa, namely, Characiformes, Gymnotiformes, and Siluriformes (table 2). Some libraries had already been sequenced by us or other research groups, and data were downloaded from the sequence read archive (SRA-NCBI), totaling six libraries (supplementary table S1, Supplementary Material online). To include five superfamilies within Characiformes (Betancur-R et al. 2019), we sequenced ten additional species on the BGISEQ-500, Illumina HiSeq or Illumina MiSeq platforms at BGI (BGI Shenzhen Corporation, Shenzhen, China) or at the Center of Functional Genomics (ESALQ/USP, Brazil; supplementary table S1, Supplementary Material online). Several studies have already demonstrated that sequencing data obtained from the BGISEQ-500 and Illumina platforms are largely comparable (Mak et al. 2017; Zhu et al. 2018; Natarajan et al. 2019; Senabouth et al. 2020), so we did not consider any possibility of platform bias. Quality checks and trimming of the adapters was performed using Trimmomatic software (Bolger et al. 2014) to remove adapter sequences and select read pairs with *Q* > 20 for all nucleotides. In total, we analyzed 16 species distributed in three orders and 11 families (fig. 1 and supplementary table S1, Supplementary Material online).

To reveal the transcription of CharSat01-52, we searched for CharSat01-52 transcripts in different samples using RNA-seq data. Here, we sequenced depleted rRNA samples from the muscle and ovaries of *A. paranae* individuals. For this experiment, all the biological samples were collected on the same day. After dissection, the tissues were immediately frozen in liquid nitrogen and stored at −70 °C. Then, RNA was extracted using the TRIzol Kit (Invitrogen) following the manufacturer’s instructions. Subsequently, the samples were treated with DNAse I and checked on 1% agarose gel with 2100 Bioanalyzer (Agilent) equipment. Only RNA samples with an A260/280 ratio of 1.8–2.0, an A260/230 ratio > 2.0, and a RIN > 7 were used for the subsequent analysis. The samples were sent to BGI (BGI Shenzhen Corporation, Shenzhen, China) and depleted with rRNA with the MGIEasy rDNA Depletion Kit before use for directional RNA-seq library preparation. Then, the samples were sequenced with the BGISEQ-500 platform (supplementary table S1, Supplementary Material online). Furthermore, we also downloaded mRNA polyadenylated RNA-seq data (SRA-NCBI) from the same sequenced tissues of *A. paranae* described above (muscle and ovaries) (Silva in preparation) and muscle of *P. mesopotamicus* (supplementary table S1, Supplementary Material online).

We evaluated the densities and lengths of CharSat01-52 arrays in two PacBio SMRT sequencing libraries for *A. paranae* and *A. mexicanus*. For the first species, we extracted DNA and checked its integrity using the HS Large Fragment 50-kb kit (Agilent). Subsequently, library preparation and sequencing on a PacBio Sequel I platform (movie time = 600 min) were performed by RTL Genomics (Research and Testing Laboratory, Lubbock, TX). In addition, we analyzed several PacBio RS II libraries of *A. mexicanus* gDNA available in the SRA of the NCBI (supplementary table S1, Supplementary Material online) with the kind permission of Dr Wesley Warren.

### Bioinformatic Protocols—Short-Read Sequencing Data

We applied distinct pipelines to determine CharSat01-52 abundance, diversity, and organization in Characiformes genomes. We subsampled 5 million read pairs (2 × 101 bp) per species for the subsequent downstream analyses. For those libraries with different read lengths, we trimmed all of the reads using Seqtk software. To investigate the tandemly repeated nature of CharSat01-52 and possible structural variations in the analyzed species, we selected pairs of reads showing homology with this satDNA by using BLAT (Kent 2002) and then created cluster graphs using RepeatExplorer (Novák et al. 2013) with at least 2 × 2,500 reads as the input.

We examined patterns in the CNV profiles of CharSat01-52 by applying the RepeatProfiler workflow (Negm et al. 2020; https://github.com/johnssproul/RepeatProfiler, last accessed August 2020). We first mapped our subsampled libraries with 5 million reads to a 208 bp-concatenated consensus monomer fragment of CharSat01-52 (MmaSat85-52, NCBI accession number MG819078.1). We also provided ten single-copy fish genes to be mapped for single-copy normalization of the read coverage (*ppfia1* [XM_022685633.1], *foxl2* [XM_007232295.3], *prospero* [XM_017708821.1], *msh4* [XM_017711771.1], *zdhhc22* [XM_017711775.1], *coq6* [XM_017711829.1], *znf106* [XM_017711848.1], *lactamase* [XM_022682177.1], *gastrula zinc finger* [XM_022685636.1], and *tubulin-kinase* [XM_017711762.1]). The mapping was performed with Bowtie2 (Langmead and Salzberg 2012); the preset values for the –sensitive and –no-mixed parameters were used. After this step, the pipeline generates color-enhanced profiles to provide a visual indication of read depth at each site of a reference sequence and allows us to test the degree of correlation in profile shape within and among groups (in our case, the within-group comparison was performed only for *C. gomesi*). The pipeline automatically applied a color ramp such that the color of all the CharSat01-52 profiles shown here indicates the copy number relative to the maximum value observed (11,435 copies in *C. gomesi*) throughout all the profiles. Furthermore, RepeatProfiler also generates variant-enhanced profiles, providing a visual summary of variant sites relative to the reference sequence.

Intra- and interspecies abundance and divergence for CharSat01-52 were also determined by RepeatMasker (Smit et al. 2017) with a cross_match search engine. After that, Kimura 2-parameter divergence values between CharSat01-52 and each of the analyzed genomes were calculated using the calcDivergenceFromAlign.pl module within the RepeatMasker suite and plotted as a repeat landscape per species (Smit et al. 2017).

To provide more direct estimates of intra- and interspecies monomer abundance and similarity, we generated an MST from CharSat01-52 monomers. First, we subsampled the short-read sequencing libraries according to the genome sizes available for each of the analyzed species (supplementary table S1, Supplementary Material online; Carvalho et al. 1998); *M. macrocephalus* was used as a starting point for selecting 1,000,000 paired reads (genome size of 1.38 Gb). Then, we extracted complete CharSat01-52 monomer sequences directly from the short-read data, discarded those sequence variants found only once (singletons) using a custom python script (https://github.com/fjruizruano/ngs-protocols/blob/master/cd_hit_filter_size.py, last accessed October 2020) and aligned the resulting data using the Muscle algorithm (Edgar 2004) under default parameters. Subsequently, this alignment file was used as input in the PHYLOViZ software (Nascimento et al. 2017) to generate an MST, as described in Utsunomia et al. (2019). In addition, these aligned monomers were displayed as separate sequence logos using WebLogo 3.3 software (Crooks et al. 2004).

BlastN searches (Altschul et al. 1990) were also carried out using consensus sequences of CharSat01-52 monomers against the nucleotide collection of the NCBI (nr database). Subsequently, we retrieved results with *e-*values lower than 1e−10. Significant alignments were produced against CharSat01-52 variants (ApaSat29-52, CgomSat02-52, MmaSat85-52, and MelSat49-52) and against PTPRF interacting protein alpha 1 (*ppfia1*) (transcript variants X1, X4, X6, X8, X10, X14, and X15). To better understand the cause of this alignment, we retrieved the genomic region of ppfia1 from the assembled genomes of the Characiformes species *A. mexicanus* (Unplaced_Scaffold 2,658 from Astyanax mexicanus-2.0) and *Pygocentrus nattereri* (Scaffold 361 from Pygocentrus_nattereri-1.0.2), the Gymnotiformes species *Electrophorus electricus* (scaffold184 from Ee_SOAP_WITH_SSPACE), the Siluriformes species *Pangasianodon hypophthalmus* (chromosome 6 from GENO_Phyp_1.0), and the Cypriniformes species *Danio rerio* (chromosome 18 from GRCz11). After that, we manually searched for the presence of CharSat01-52.

RNA-seq reads were mapped to a 208-bp-concatenated consensus monomer fragment of CharSat01-52 and also to four endogenous genes, namely: 1) *rpl13a* (accession number: XM_007244599.3), 2) *rpl32* (accession number: XM_007251493.2), 3) *rpl8* (accession number: XM007227850.3), and 4) *hprt* (accession number: XM_022684242.1) using Bowtie2 (Langmead and Salzberg 2012) with the preset values for the –sensitive and –no-mixed parameters. Then, the mapping data were converted into a sorted binary format using SAMtools (Li et al. 2009). Subsequently, we extracted the number of mapped reads with a custom script (https://github.com/fjruizruano/ngs-protocols/blob/master/bam_coverage_join.py, last accessed October 2020) and estimated their transcription level as FPKM (fragments per kilo-base of transcript per million reads mapped). The values are presented as the mean ± SD. Furthermore, we subsampled the RNA-seq libraries (32,000,000 paired-end reads), isolated monomers directly from raw reads and constructed an MST together with extracted monomers from the genomic libraries of *A. paranae* (subsample of 32,000,000 paired-end reads) and *P. mesopotamicus* (sample of 4,914,670 paired-end reads) (supplementary table S2, Supplementary Material online).

### Bioinformatic Protocols—Long-Read Sequencing Data

Short-read data can only provide information regarding total repeat abundances and the tandemly repeated nature of satDNAs. Therefore, we used long reads in conjunction with FISH analyses to provide a broader genomic panorama of *A. paranae* and *A. mexicanus*. Considering the error-prone nature of PacBio CLR technology (∼15% error rates; Rhoads and Au 2015), repeated motifs were identified in PacBio subreads using NCRF version 1.01.00 (Harris et al. 2019). The –*maxnoise* parameter was set to 20% to retain long reads with noisy repeat arrays, as described in Cechova et al. (2019). To test the reliability of our data, we searched in the PacBio libraries for the sequences of two satDNAs with different FISH patterns: 1) CharSat01-52, which is organized as small tandem arrays in *A. paranae* and *A. mexicanus* and 2) AmeSat02-179 and ApaSat10-179 (NCBI accession number: MF044776.1), which are homologous and consistently clustered in both species in the pericentromeric regions, as demonstrated in a previous study (Utsunomia et al. 2017). Subsequently, the CharSat01-52 and AmeSat02-179/ApaSat10-179 repeat densities were calculated as the total number of kilobases annotated per million sequenced bases (kb/Mb).

We also applied distinct pipelines to search for the recurrent association of CharSat01-52 arrays with other repeats, such as transposable elements and satDNAs. First, we applied a custom python script (https://github.com/MilanCalegari/FlankerExtractor, last accessed November 2020) to select 10-kb regions upstream and downstream of every CharSat01-52 locus identified with NCRF; when the corresponding adjacent region of the read did not reach 10 kb, we analyzed the read up to the end. At this point, we applied two distinct pipelines to this subset of sequences: 1) We constructed a custom database composed of the transposable elements identified in the genome of *A. mexicanus* (http://www.fishtedb.org/project/download?species=Astyanax±mexicanus, last accessed August 2020) and the satellitome of *A. paranae* (Silva et al. 2017). Then, we performed a search of these sequences with LASTZ (Harris 2007) in our subset and summarized the frequencies of the distinct types of TEs/satDNAs detected within the 10-kb window (Vondrak et al. 2020). 2) Considering that the results obtained by applying the abovementioned approach are biased to sequences present in our database, we also clustered our subset of sequences using CD-HIT (Li and Godzik 2006) with a minimum cluster size of 3 and a similarity threshold of 0.8. Such a method would cluster recurrent sequences associated with CharSat01-52 arrays that could be searched against different databases (the nr database of the NCBI and the giri REPBASE, for example). Finally, we used FlexiDot (Seibt et al. 2018) to generate dot plots for studying the structure of the CharSat01-52 arrays in the PacBio reads. In these dot plots, we highlighted the presence of *A. paranae* satDNAs (Silva et al. 2017).

### Relative Quantification of Genomic Abundance and Transcription Analysis of CharSat01-52

Quantification of relative copy number of CharSat01-52 was carried out in the referred species in supplementary table S3, Supplementary Material online, through qPCR. The relative quantification of CharSat01-52 was assessed by using the 2^-ΔC^^*t*^ method (Bel et al. 2011 ) using the single-copy gene hypoxanthine phosphoribosyltransferase (*hprt1*) as reference. Primers for this gene were designed with Primer3 (Untergasser et al. 2012). The reactions were performed using SYBR Green PCR Master Mix (Thermo Fisher Scientific). Target and reference sequences were simultaneously analyzed in triplicate for three independent samples. The specificity of the PCR products was confirmed by dissociation curve analysis. The values are presented as the mean ± SD.

Transcription of CharSat01-52 was separately analyzed in the muscle and gonads of *A*. *paranae* and *C. gomesi*. In this case, cDNA of each sample was first synthetized using the High-Capacity cDNA Reverse Transcription Kit (Thermo Fisher Scientific) with 100 μg per sample of total RNA, following the manufacturer’s instructions. After that, the RT-qPCR followed the same parameters as the qPCR detailed above, except for using cDNA instead of gDNA. Here, we also chose the level of *hprt* expression as reference mRNA control. Target and reference sequences were simultaneously analyzed in multiple replicates (fig. 4 and supplementary table S3, Supplementary Material online). Relative gene expression profiles were calculated using the 2^-ΔΔC^^*t*^ method (Livak and Schmittgen 2001).

### Molecular and Cytogenetic Analyses

A primer pair was previously designed for CharSat01-52 in *A. paranae* (ApaSat29-52F and ApaSat29-52R primers, see Silva et al. 2017). We verified that this primer pair anchors in a conserved region of the monomers (fig. 2) and then used them to amplify CharSat01-52 using PCR in all our Characiformes species. The PCRs contained 1× PCR buffer, 1.5 mM of MgCl_2_, 200 μM of each dNTP, 0.1 μM of each primer, 2–100 ng of genomic DNA, and 0.5 U of Taq polymerase in a total volume of 25 μl. The PCR program consisted of an initial denaturation at 95 °C for 5 min, followed by 35 cycles at 95 °C for 10 s, 56 °C for 15 s, 72 °C for 10 s, and a final extension at 72 °C for 15 min. The PCR products were checked in 2% agarose gels. Next, we generated DNA probes for CharSat01-52 using these PCR products for all the species, except *H. malabaricus* (for which PCR failed) and labeled the probes with digoxigenin-11-dUTP or biotin-16-dUTP. This procedure allowed us to perform FISH for distinct species using probes obtained from their own genomes. In addition, we produced biotin-labeled probes of AmeSat02-179/ApaSat10-179 (NCBI accession number: MF044776.1), because this satDNA is highly clustered in the genomes of *A. mexicanus* and *A. paranae* (fig. 5*C*) (Utsunomia et al. 2017). For this reason, it could be used as a parameter to the array sizes of CharSat01-52. FISH was performed under high-stringency conditions using the method described by Pinkel et al. (1986) with small modifications that were described in Utsunomia et al. (2017). Since FISH signals were not detected in several species (supplementary fig. S5, Supplementary Material online), we performed two rounds of signal amplification using conjugated antiavidin-biotin. Each round consisted of incubating the slides for 30 min in a moist chamber at 37 °C with the amplification mix, containing 2.5% antiavidin-biotin conjugate in blocking buffer (5% nonfat dry milk in 4 × SSC), washing the slides three times in 4 × SSC, 0.5% Triton for 3 min each, then incubating the slides for 30 min in a moist chamber at 37 °C in the avidin-FITC solution (containing 0.07% avidin-FITC conjugate in blocking buffer). From each individual, a minimum of ten cells was analyzed for FISH.

## Supplementary Material

Supplementary data are available at *Genome Biology and Evolution* online.

## Supplementary Material

evab002_Supplementary_Data

## Data Availability

The data underlying this article are available in the GenBank Nucleotide Database and can be accessed with the following accession numbers: MmaSat85-52 (MG819078.1), *ppfia1* (XM_022685633.1), *foxl2* (XM_007232295.3), *prospero* (XM_017708821.1), *msh4* (XM_017711771.1), *zdhhc22* (XM_017711775.1), *coq6* (XM_017711829.1), *znf106* (XM_017711848.1), *lactamase* (XM_022682177.1), *gastrula zinc finger* (XM_022685636.1), *tubulin-kinase* (XM_017711762.1), *rpl13a* (XM_007244599.3), *rpl32* (XM_007251493.2), *rpl8* (XM007227850.3), and *hprt* (XM_022684242.1). Whole-genome sequencing data are also available in the sequence read archive (SRA) and the accession numbers for all the analyzed libraries are indicated in supplementary table S1, Supplementary Material online.
