# Supplementary material for: A Long-Term Conserved Satellite DNA That Remains Unexpanded in Several Genomes of Characiformes Fish Is Actively Transcribed
Source: Genome Biol Evol. 2021 Jan 27;13(2):evab002. doi: 10.1093/gbe/evab002 (PMC8210747; doi:10.1093/gbe/evab002)
Supplement: evab002_Supplementary_Data [file evab002_supplementary_data.zip › Supplementary Material_all.pdf]

Figure S1 – Supplementary material

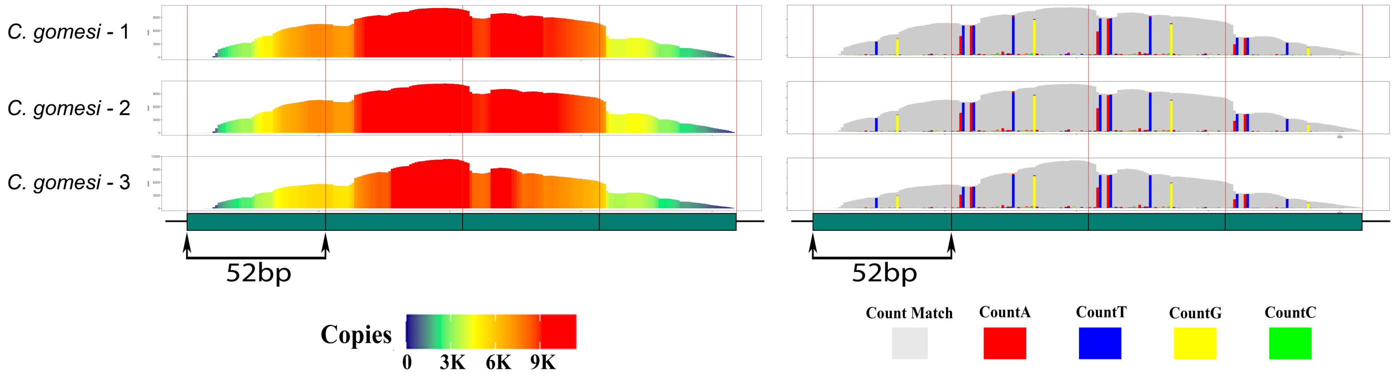

Fig. S1: Intraspecific CNV profiles for CharSat01-52 in different samples of *C. gomesi*. Note the similar CNV and variant profiles for different samples.

Figure S2 – Supplementary material

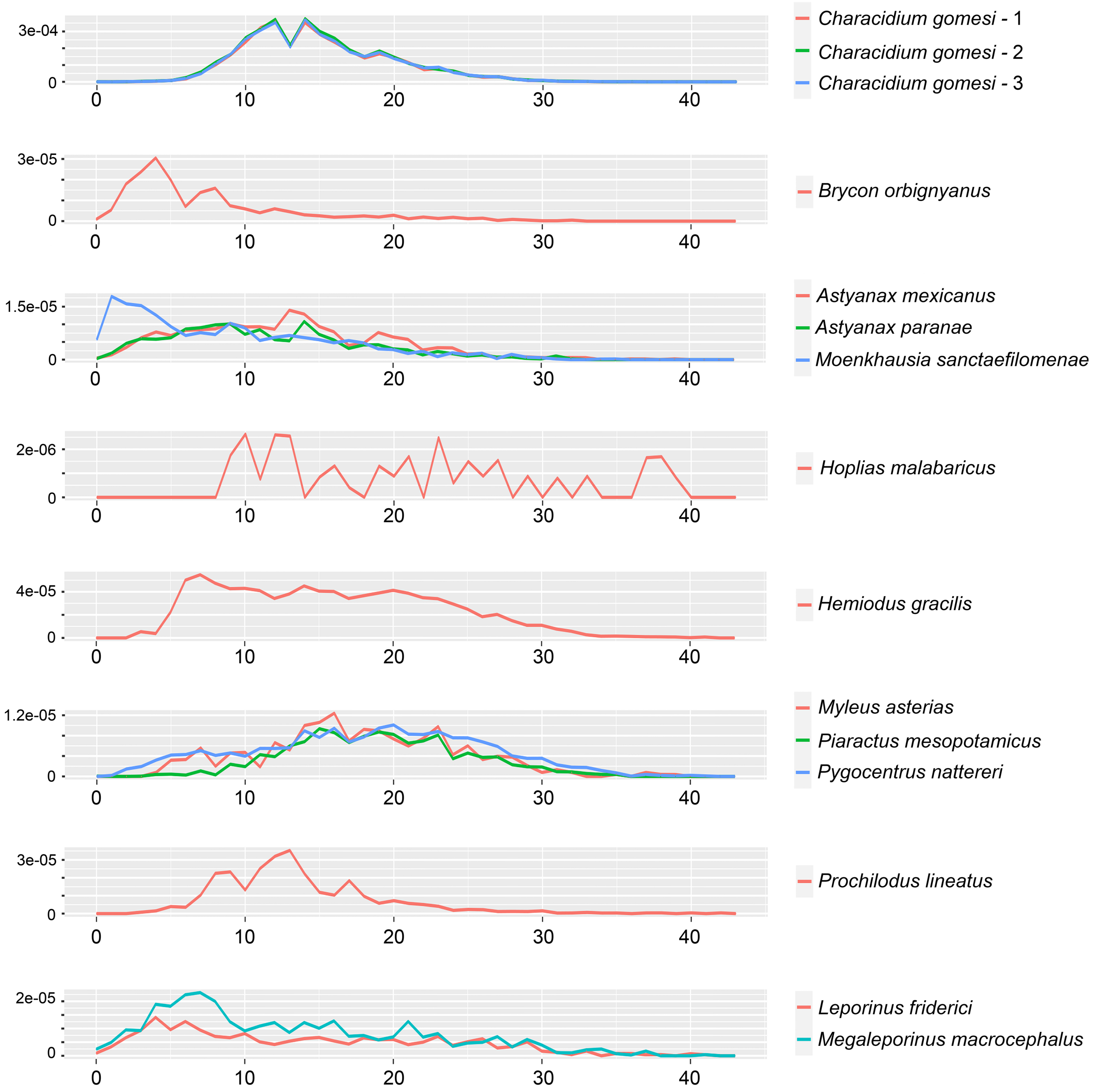

Fig. S2: Repeat landscapes showing the genomic abundance (Y-axis) and Kimura divergence profiles (X-axis) for CharSat01-52 in several Characiformes, suborder Characoidea species grouped by family. In this case, Crenuchidae, Bryconidae, Characidae, Erythrinidae, Hemiodontidae, Serrasalmidae, Prochilodontidae and Anostomidae. Note the similarity among samples from the same family.

Figure S3 – Supplementary material

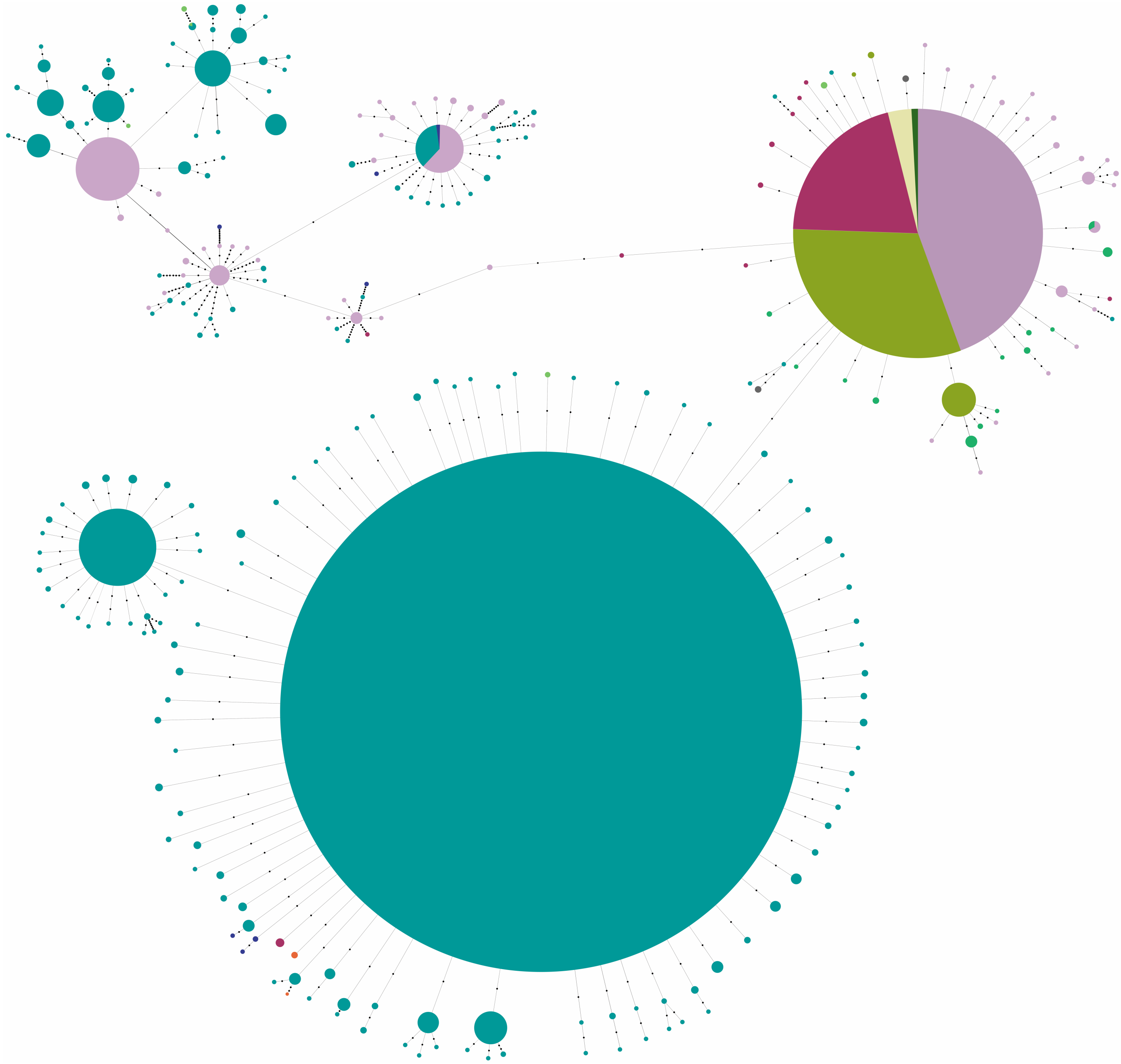

Fig. S3: Minimum spanning tree showing the relationships between the isolated monomers obtained from distinct species. Colored circles represent monomers retrieved from Illumina reads and the diameter of the circles is proportional to their abundance (linear scale).

Figure S4 – Supplementary material

Astyanax mexicanus (Characiformes) – *ppfia1*

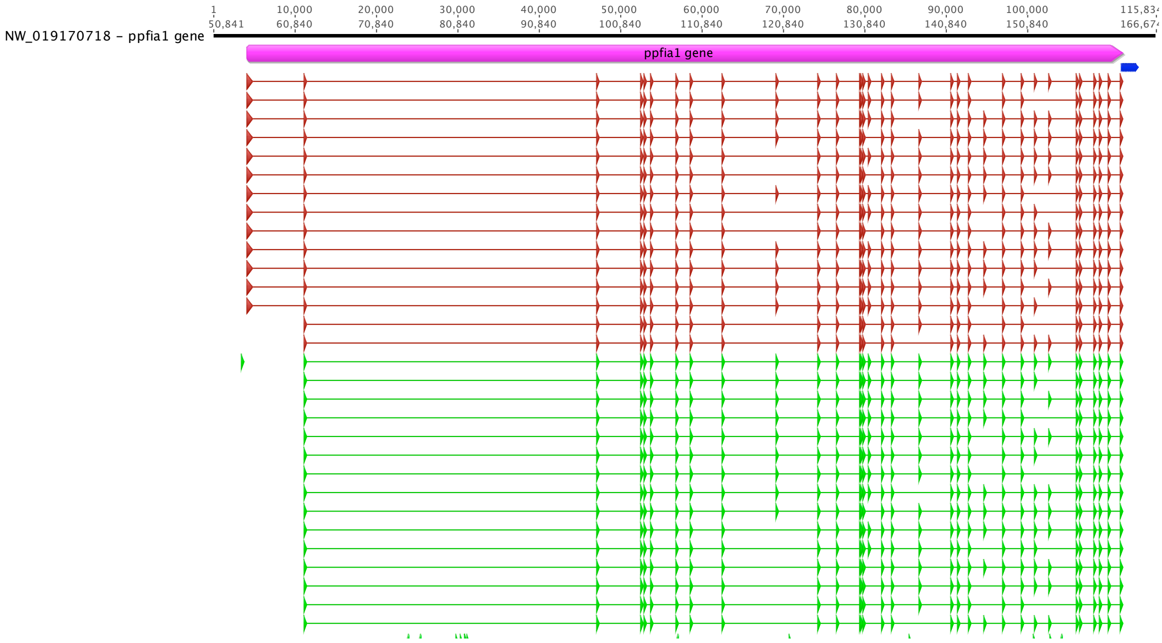

Astyanax mexicanus (Characiformes) – *ppfia1* (zoom in)

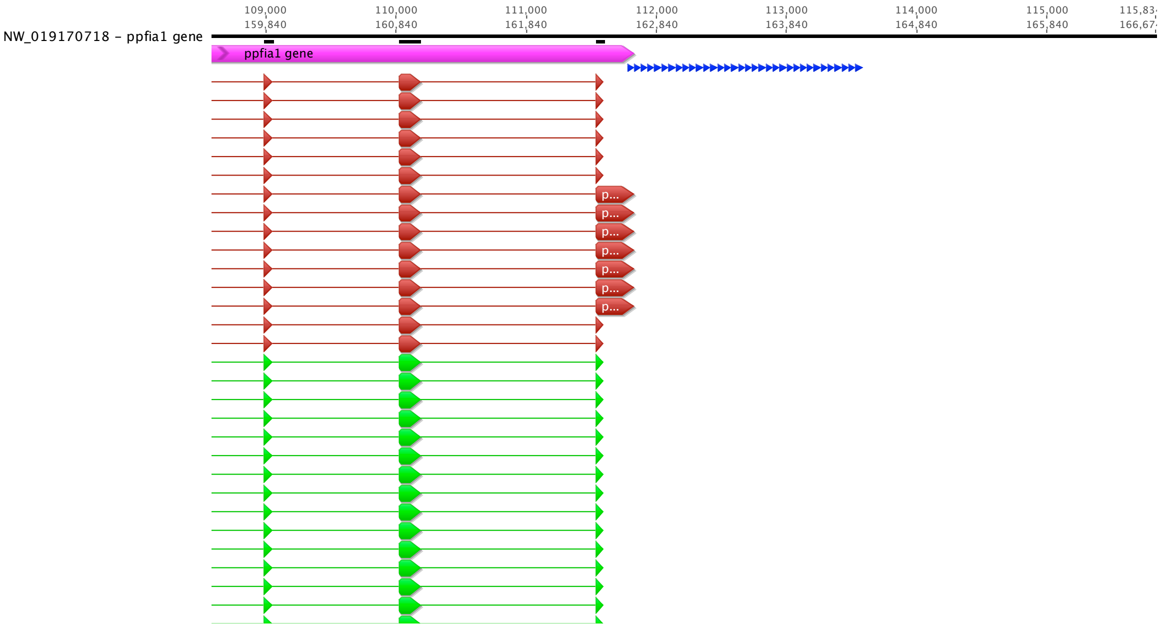

Pygocentrus nattereri (Characiformes) – *ppfia1*

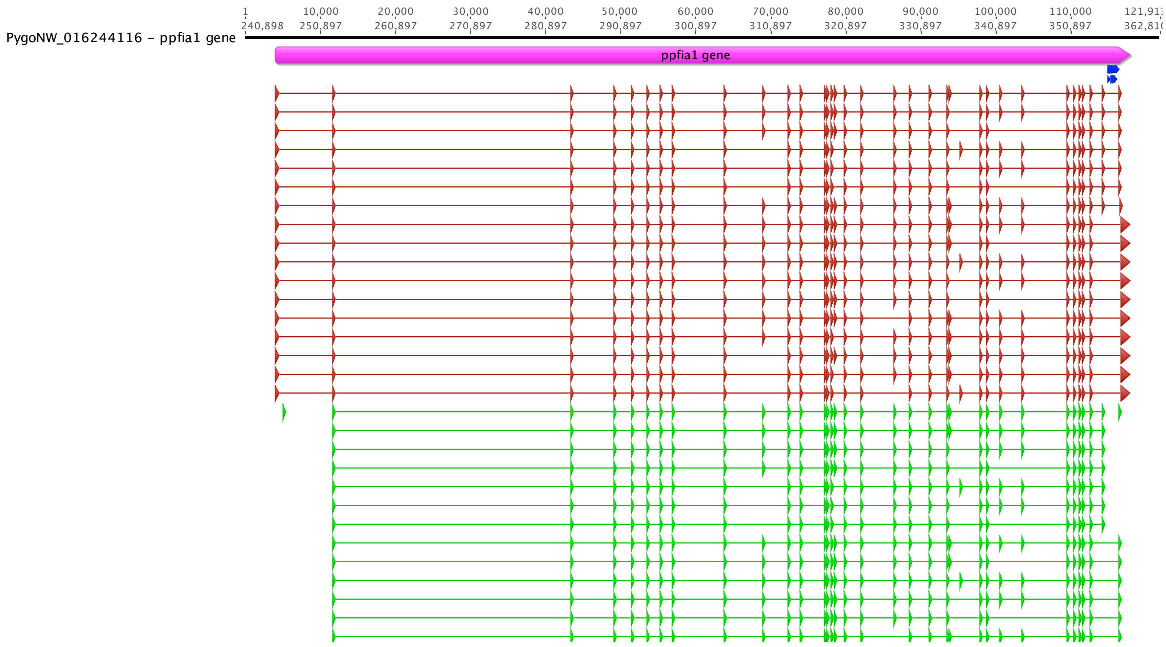

Pygocentrus nattereri (Characiformes) – *ppfia1* (zoom in)

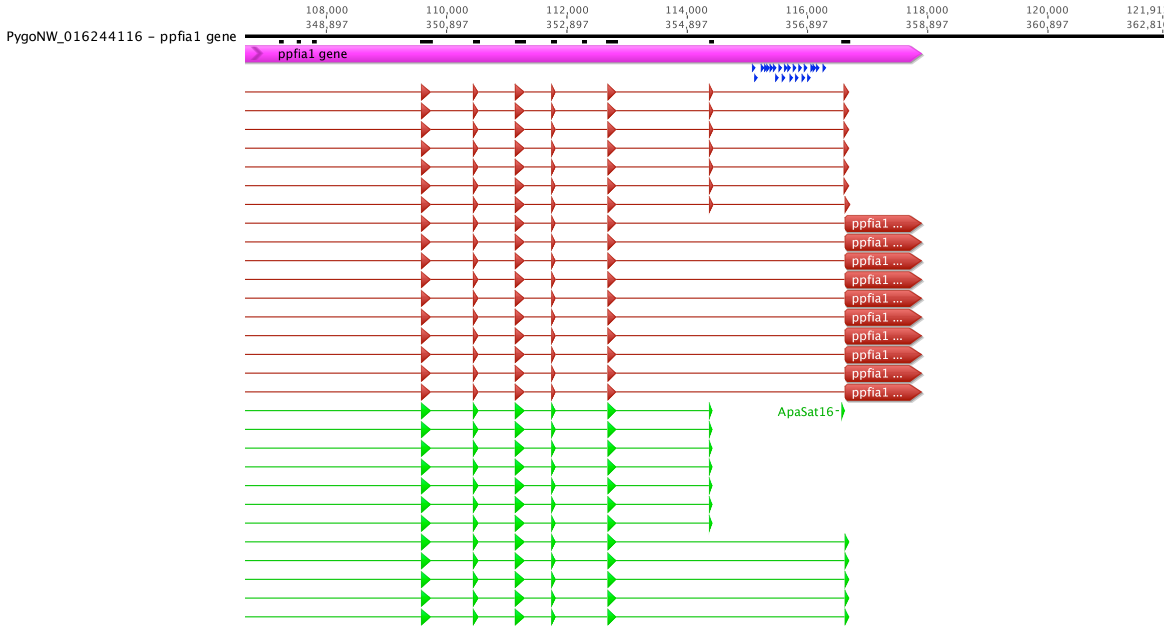

Pangasianodon hypophthalmus (Siluriformes) – *ppfia1*

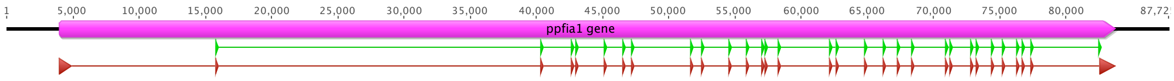

Electrophorus electricus (Siluriformes) – *ppfia1*

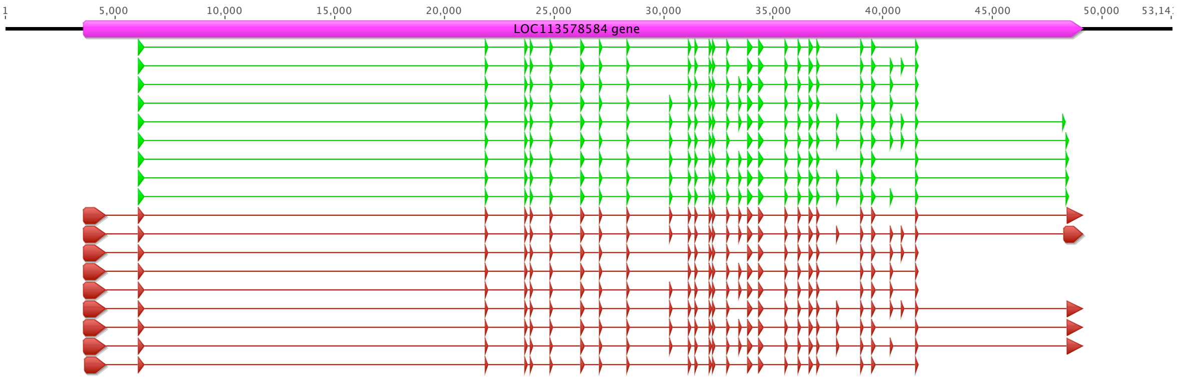

Danio rerio (Cypriniformes) – *ppfia1*

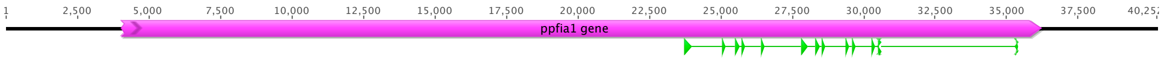

Fig. S4: *ppfia1* gene structure and transcripts in several Otophysa species. Note the presence of annotated CharSat01-52 (blue triangles) only in Characiformes species (*A. mexicanus* and *P. nattereri*). In *A. mexicanus*, the satellite is located on the 3'UTR, while in *P. nattereri*, the satellite is located in the last intron.

Figure S5 – Supplementary material

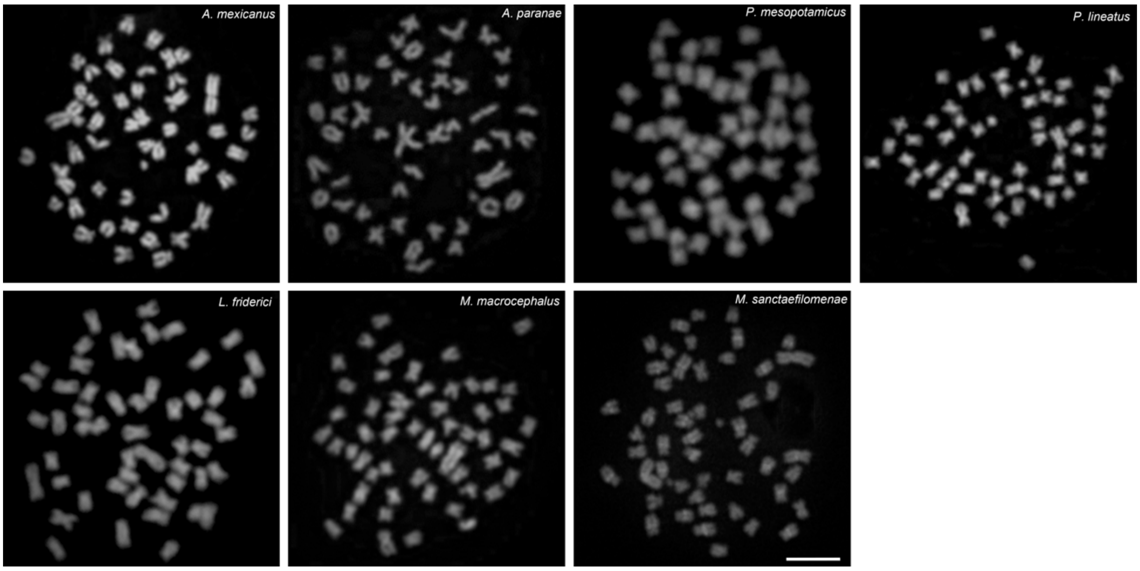

Fig. S5: Distribution of CharSat01-52 on the metaphase chromosomes of several Characoidea species without performing signal enhancement. Bar =10 μm

Figure S6 – Supplementary material

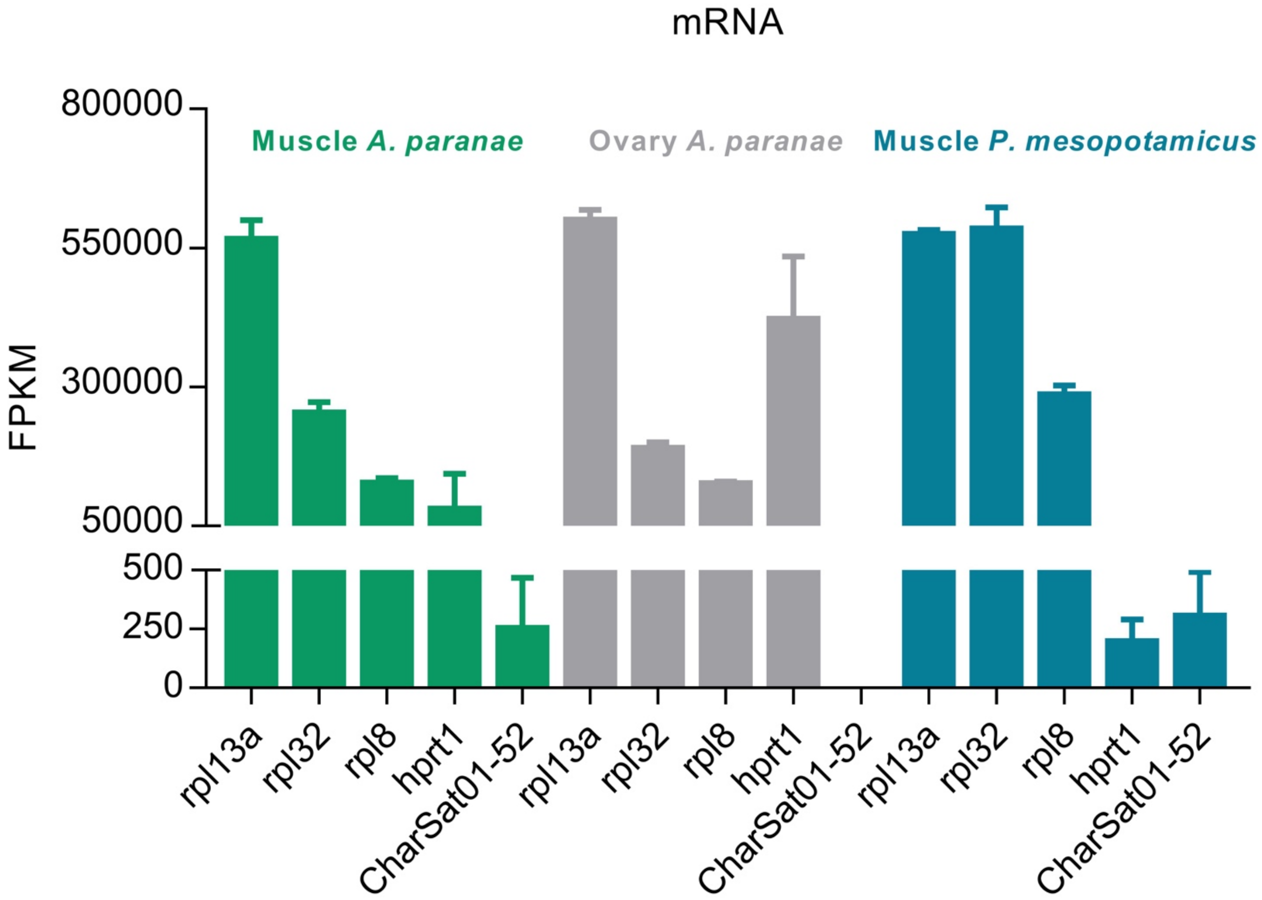

Fig. S6: Transcription levels of CharSat01-52 in different mRNA-seq libraries. Expression was measured as FPKM.
