# Supplementary material for: A Long-Term Conserved Satellite DNA That Remains Unexpanded in Several Genomes of Characiformes Fish Is Actively Transcribed
Source: Genome Biol Evol. 2021 Jan 27;13(2):evab002. doi: 10.1093/gbe/evab002 (PMC8210747; doi:10.1093/gbe/evab002)
Supplement: evab002_Supplementary_Data [file evab002_supplementary_data.zip › Supporting information legends.docx]

**Supporting information legends**

**Fig. S1:** Intraspecific CNV profiles for CharSat01-52 in different samples of *C. gomesi*. Note the similar CNV and variant profiles for different samples.

**Fig. S2:** Repeat landscapes showing the genomic abundance (Y-axis) and Kimura divergence profiles (X-axis) for CharSat01-52 in several Characiformes, suborder Characoidea species grouped by family. In this case, Crenuchidae, Bryconidae, Characidae, Erythrinidae, Hemiodontidae, Serrasalmidae, Prochilodontidae and Anostomidae. Note the similarity among samples from the same family.

**Fig. S3:** Minimum spanning tree showing the relationships between the isolated monomers obtained from distinct species. Colored circles represent monomers retrieved from Illumina reads and the diameter of the circles is proportional to their abundance (linear scale).

**Fig. S4:** *ppfia1* gene structure and transcripts in several Otophysa species. Note the presence of annotated CharSat01-52 (blue triangles) only in Characiformes species (*A. mexicanus* and *P. nattereri*). In *A. mexicanus*, the satellite is located on the 3’UTR, while in *P. nattereri*, the satellite is located in the last intron.

**Fig. S5:** Distribution of CharSat01-52 on the metaphase chromosomes of several Characoidea species without performing signal enhancement. Bar =10 μm

**Fig. S6:** Transcription levels of CharSat01-52 in different mRNA-seq libraries. Expression was measured as FPKM.
